# Birth Preparedness and Complication Readiness (BPCR) interventions to reduce maternal and neonatal mortality in developing countries: systematic review and meta-analysis

**DOI:** 10.1186/1471-2393-14-129

**Published:** 2014-04-04

**Authors:** Dieudonné Soubeiga, Lise Gauvin, Marie A Hatem, Mira Johri

**Affiliations:** 1 Department of Health Administration, Faculty of Medicine, University of Montreal, Montreal, Canada; 2 Department of Social and Preventive Medicine Faculty of Medicine, University of Montreal, Montreal, Canada; 3 Division of Global Health, University of Montreal Hospital Research Centre (CRCHUM), Montreal, Canada

**Keywords:** Birth preparedness, Neonatal mortality, Maternal mortality, Antenatal education, Women’s groups, Community mobilization, Behavior change, Developing countries, Meta-analysis

## Abstract

**Background:**

Birth Preparedness and Complication Readiness (BPCR) interventions are widely promoted by governments and international agencies to reduce maternal and neonatal health risks in developing countries; however, their overall impact is uncertain, and little is known about how best to implement BPCR at a community level. Our primary aim was to evaluate the impact of BPCR interventions involving women, families and communities during the prenatal, postnatal and neonatal periods to reduce maternal and neonatal mortality in developing countries. We also examined intervention impact on a variety of intermediate outcomes important for maternal and child survival.

**Methods:**

We conducted a systematic review and meta-analysis of randomized trials of BPCR interventions in populations of pregnant women living in developing countries. To identify relevant studies, we searched the scientific literature in the Pubmed, Embase, Cochrane library, Reproductive health library, CINAHL and Popline databases. We also undertook manual searches of article bibliographies and web sites. Study inclusion was based on pre-specified criteria. We synthesised data by computing pooled relative risks (RR) using the Cochrane RevMan software.

**Results:**

Fourteen randomized studies (292 256 live births) met the inclusion criteria. Meta-analyses showed that exposure to BPCR interventions was associated with a statistically significant reduction of 18% in neonatal mortality risk (twelve studies, RR = 0.82; 95% CI: 0.74, 0.91) and a non-significant reduction of 28% in maternal mortality risk (seven studies, RR = 0.72; 95% CI: 0.46, 1.13). Results were highly heterogeneous (I^2^ = 76%, p < 0.001 and I^2^ = 72%, p = 0.002 for neonatal and maternal results, respectively). Subgroup analyses of studies in which at least 30% of targeted women participated in interventions showed a 24% significant reduction of neonatal mortality risk (nine studies, RR = 0.76; 95% CI: 0.69, 0.85) and a 53% significant reduction in maternal mortality risk (four studies, RR = 0.47; 95% CI: 0.26, 0.87).

Pooled results revealed that BPCR interventions were also associated with increased likelihood of use of care in the event of newborn illness, clean cutting of the umbilical cord and initiation of breastfeeding in the first hour of life.

**Conclusions:**

With adequate population coverage, BPCR interventions are effective in reducing maternal and neonatal mortality in low-resources settings.

## Background

In spite of important progress towards attaining the Millennium Development Goals (MDGs), maternal and neonatal mortality continue to figure as major public health problems in developing countries [1,2]. Improvements in maternal health and reductions in maternal mortality have been slower than anticipated and – despite isolated successes – remain far from the MDG5 target of a 75% reduction in the maternal mortality ratio (MMR) from 1990 to 2015 [3]. Although child survival progress is accelerating [4], only 31 countries are on track to achieve the MDG4 target to reduce child mortality by two-thirds between 1990 and 2015 [2]. Moreover, over the period 2000–2010 decreases in mortality have been more rapid in the age group 1–59 months, such that the neonatal fraction of deaths has increased from 38.2% to 40.3% [4]. To achieve MDGs 4 and 5, the global community will need to focus attention and resources on effective strategies to reduce maternal and neonatal deaths, particularly in poor and underserved communities [5].

Developing countries have recently invested in behavior change and community mobilisation interventions to reduce maternal and neonatal risks following the concept of “Birth Preparedness and Complication Readiness” (BPCR), which comprises elements of antenatal, intrapartum, postpartum care and neonatal care [6]. BPCR programs generally include counselling for women and their families to: 1) encourage them to take decisions before the onset of labour and potential occurrence of obstetric complications; 2) inform them about the signs of complications so they will know and be able to react promptly if needed; 3) inform them about the locations of emergency services to make the care-seeking process more efficient; and 4) encourage them to save the money needed to pay for services and to plan their transportation to a health facility during labour and in case of emergency [6-9].

To aid in BCPR implementation, the Johns Hopkins Program for International Education in Gynecology and Obstetrics (JHPIEGO) has developed a BPCR matrix [6] that delineates the roles of policymakers, facility managers, providers, communities, families, and women in ensuring that women and newborns receive appropriate, effective, and timely care. The BPCR matrix outlines plans and actions that can be implemented by each group of stakeholders to build an enabling environment for normal and emergency care.

BCPR is a broad and integrative strategy; evidence related to its comprehensive implementation is scarce. However, components of the BPCR matrix have been implemented and evaluated in many settings [10-14]. BPCR components are included in the new World Health Organization (WHO) model for antenatal care as part of antenatal care education in clinic setting [15]. Based on critical primary research in India [16] and elsewhere, WHO and UNICEF [17] also now recommend antenatal and postnatal home visits to counsel mothers, provide newborn care and facilitate referral [18]. In addition to making use of formal health services, BPCR requires making effective use of community health workers and health promotion groups. A 2010 systematic review and meta-analysis of community-based intervention packages found a significant reduction in neonatal mortality (twelve studies, risk ratio 0.76, 95% CI 0.68, 0.84), but inconclusive evidence of reduction in maternal mortality (ten studies, risk ratio 0.77; 0.59, 1.02) [19]. Community mobilization through stakeholders such community health workers, or through participation in women’s groups also forms part of the BPCR concept [20]. This component was recently evaluated in a Lancet systematic review and meta-analysis focussing on trials involving women’s groups practising participatory learning and action [21]. Meta-analyses of seven trials showed that exposure to women’s groups was associated with a 37% reduction in maternal mortality (odds ratio 0.63, 95% CI 0.32, 0.94), a 23% reduction in neonatal mortality (odds ratio 0.77; 0.65, 0.90).

This systematic review and metanalysis provides the first assessment of the full range of BPCR strategies on maternal mortality, neonatal mortality, and a variety of intermediate outcomes critical for maternal and child survival. It also aims to assess which components of the BPCR concept are most effective.

### Objective of the review

The primary aim of this review was to evaluate the impact BPCR interventions in reducing maternal and neonatal mortality in developing country settings. We also examined the impact of BPCR interventions on process outcomes such use of skilled services, and hygienic practices in the home. Stratified analyses were used to examine program impact in relation to types of interventions and background neonatal mortality level.

## Methods

### Criteria for including studies in the review

#### *Types of studies*

We considered only randomized trials. The unit of randomization could be at the individual or cluster level.

#### *Participants*

Participants were pregnant women who received BPCR interventions and lived in developing countries as classified by the World Bank [22].

#### *Types of interventions*

These were intervention packages that included any component of the BPCR concept, individually or in combination. Interventions could take place in antenatal, intrapartum, postpartum and neonatal care periods; and at different levels of care (provider, facility, home, community). Specific approaches assessed included counselling of women in prenatal clinics, home visit strategies; and community mobilisation activities.

#### *Comparator group*

Women who received no experimental BPCR intervention defined by studied trial.

#### *Outcome measures*

Primary outcomes are maternal mortality and neonatal mortality. Secondary outcomes are institutional delivery, home delivery with skilled birth attendant, use of skilled care for neonatal illness, use of postpartum care, clean cutting of the umbilical cord, initiation of breastfeeding within the first hour of birth, knowledge of maternal and neonatal danger signs, and birth preparedness and complication readiness behaviours.

#### *Language of publication*

Only studies published in English or French were considered.

### Search methods to identify studies

The search strategy was designed in conjunction with an information retrieval specialist and followed Cochrane collaboration guidelines [23]. We searched the PubMed, Embase, Cochrane library, Reproductive Health library, POPLINE and CINAHL databases. The date of search was December 17th, 2012, updated December 5th 2013. The search strategy combined the terms “Birth preparedness”, “antenatal education”, “home visits”, “Community mobilisation”, “women’s groups” “maternal mortality”, “neonatal mortality” “facility-based childbirth” and “developing countries” (Additional file 1 presents a sample search strategy). To supplement the electronic searches, we also hand-checked bibliographies of review papers and related articles [21], international agency websites (WHO, UNFPA, JHPIEGO, USAID and CARE) and two scientific journals specialized in maternal and neonatal health: *BMC Pregnancy and Childbirth* and *International Journal of Gynaecology & Obstetrics*.

### Selection of studies

Two authors (DS and MJ) reviewed titles, abstracts and keywords of all articles retrieved by the search strategy. Studies that did not meet criteria related to type of study, participants, intervention, and study country were excluded. The full texts of candidate studies were then examined; those that did not meet inclusion criteria were discarded.

### Data extraction

Two authors (DS, MJ) extracted data on the interventions, participants, outcomes and findings, as well as on indicators of methodological quality (randomization, blinding methods, losses to follow-up, etc.). Authors jointly determined study inclusion on the basis of their individual assessments and discussion.

### Assessment of methodological quality

Three authors (DS, MJ and LG) assessed the methodological quality of the included studies. We established nine criteria for methodological quality based on the recommendations of the Cochrane Collaboration [24] and the McMaster Quality Assessment Tool for Quantitative Studies [25]. Each dimension was rated *adequate*, *inadequate*, or *unclear*, based on the information reported.

1. *Randomization*. The method used to generate the allocation sequence was rated adequate if the procedure used was genuinely random (random number table, software, etc.), inadequate if the procedure was not random, or unclear if the information was missing.

2. *Concealment of the allocation sequence. Adequate* methods to prevent selection bias are, for example, centralized randomization and the use of opaque and sealed numbered envelopes.

3. *Blinding of evaluators* was rated *adequate* if the study used independent evaluators who were blind to the intervention.

4. *Contamination* was rated *adequate* if the steps taken to prevent the control group from receiving the intervention were described.

5. *Co-intervention* was rated *adequate* if the article mentioned the absence of any additional intervention in the intervention or control groups.

6. *Coverage*. In the trial conducted by Azad et al. [26], the authors considered a participation rate in group sessions of at least 30% of pregnant women as the minimum necessary to achieve desired results. Thus, we considered coverage above a threshold of 30% as *adequate*.

7. *Quality of implementation*. This component refers to measures taken to ensure that the intervention was administered as planned (i.e., training of educators, use of practice guidelines, supervision, etc.). This component was rated *adequate* if the article mentioned specific training for the implementation staff or the use of practice guidelines for the education sessions.

8. *Losses to follow-up*. For individual trials, this component was rated *adequate* if at least 90% of the participants completed the study. For cluster trials, an *adequate* rating meant that no cluster was lost.

9. *Analysis based on “intention to treat” (ITT)*.

Quality assessments did not influence inclusion of studies in the meta-analyses. However, these assessments later served as criteria for subgroup analyses, and were used in interpreting results.

### Data synthesis

We performed meta-analyses to combine relative risks (RR) comparing intervention groups with control groups. Meta-analyses used a random effects model due to important variations in populations and in interventions. Combined RRs and 95% confidence intervals (CIs) were calculated for outcomes measured in the same way by at least two studies. All were binary variables. The number of studies contributing to the meta-analyses ranged from two to 12. Data were re-analyzed based on the ITT principle and baseline differences in outcomes were assumed to have little influence. Combinations were carried out using the Mantel-Haenszel method in the Cochrane Review Manager software [27]. For results reported as cluster averages, the number of events for each group was estimated using the formula N*cluster average/100.

To adjust for cluster effects, for each study randomized in clusters we divided the original number of participants by the cluster effect, whose value was 1 + (M-1)*ICC, where M was the average cluster size and ICC, the intraclass correlation coefficient [24].

Finally, we prepared a description of the reported results for outcomes not included in the meta-analyses, such as knowledge of maternal and neonatal danger signs and birth preparation behaviours.

### Investigation of heterogeneities and subgroup analyses

To investigate heterogeneities we calculated the I^2^ statistic, which describes the percentage of total variation among studies due to heterogeneity rather than to chance [28]. An I^2^ value of 50% or more indicates significant heterogeneity among studies.

Subgroup analyses were planned on the basis of factors identified a priori as potential sources of heterogeneity. These were: methodological quality of the trials; place of intervention (i.e., prenatal clinic, home or community); intervention approach (i.e., clinic-based counselling, home visit strategy, community mobilization led by stakeholders and women ‘groups participatory sessions); participants’ living environment (i.e., rural or urban); baseline or control group neonatal mortality rate (i.e., ≤ 30‰; > 30‰ to < 40‰; ≥ 40‰); baseline or control group facility-based delivery rate (i.e., <30%; ≥ 30% to < 50%; ≥ 50% to < 70%; ≥ 70%); components of the intervention (i.e., only prenatal education, both prenatal and postnatal education); and involvement of people from the woman’s social network (i.e., husband, other family member, or member of the community). However, there were not enough studies to cover the different subgroup modalities and only intervention type, background neonatal mortality rate and women’s participation rate could ultimately be analysed.

## Results

### Results of the initial search strategy

**Figure 1:**
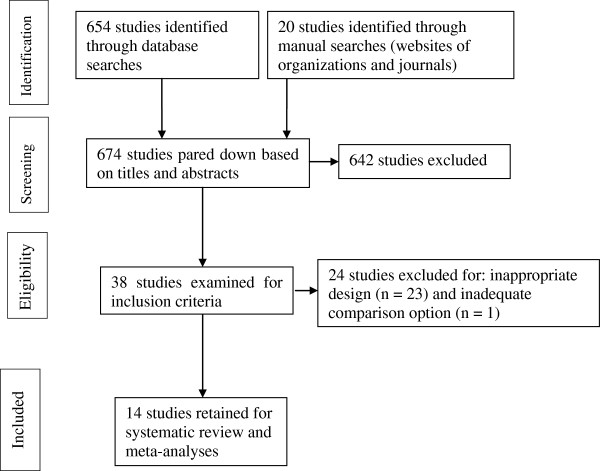
Flow diagram for the selection of studies.

Electronic and manual searches identified 654 potentially useful reports, after elimination of duplicates (see Figure 1). We examined titles and abstracts of these 654 studies and 38 reports were retained for full text review.

### Description of studies included in the review

The Additional file 2 describes the 14 randomized studies retained. Two studies [29,30] used individual randomized units. The other 12 were cluster trials with geographic entities (villages, administrative unions or neighbourhoods) as the randomization units.

Study settings were Indi, Nepal , Bangladesh, Ghana, Malawi, Pakistan and four Latin American cities (Rosario in Argentina, Pelotas in Brazil, Havana in Cuba, and Mexico City in Mexico).

#### *Characteristics of the interventions*

*Objectives*. To assess the impact of educational interventions and community mobilization on neonatal mortality [31,32]; to test the impact of the husband’s involvement in prenatal education on the use of maternal care services and birth preparation [29]; to measure the effectiveness of the women’s groups program in addressing maternal and neonatal care [33]; and to show whether an intervention providing education and psychological support to pregnant women could change health behaviours and service utilization [30].

*Participants*. For all interventions, the target population consisted of pregnant women. Belizan’s study [30] selected prenatal care attendees presenting at least one of eight predefined risk factors. In addition to pregnant women, studies included husbands [29], persons close to the women [30], other women of reproductive age in the community [20,26,33], or community leaders [32].

*Type of interventions*. The 12 randomized cluster studies evaluated a whole series of interventions including prenatal and postnatal components. Only in the two individual trials assessing individual counselling in prenatal clinics were the interventions purely prenatal. Three studies considered a home visit strategy. Seven studies involved participation in women’s groups engaged in action-learning cycles. Two studies combined community mobilisation with home visits.

In the women’s groups approach, the implementation workers acted as *facilitators* and organized monthly meetings with each group, set up on the basis of neighbourhood proximity. In these meetings, the facilitators guided the women through the four phases of the action-learning cycle: identifying and prioritizing problems, planning strategies, implementing strategies and evaluating the effects. In this way, the women were encouraged to develop actions based on their perceptions of maternal and neonatal issues. Each group was free to implement its own combination of action [20].

#### *Outcomes measured*

The Additional file 2 provides an overview of the outcomes measures reported in the 14 studies. All studies measured multiple outcomes. Neonatal mortality was the main outcome measured in 12 cluster-randomised trials. Maternal mortality was assessed in seven studies.

#### *Methodological quality of the retained studies*

As a whole, the methodological quality of the studies was acceptable (see Additional file 3). In all studies, randomization, co-intervention, quality of implementation, losses to follow-up, and analysis based on intention to treat (ITT) were rated as *adequate*. Evaluator blinding was rated *inadequate* in all studies, except for three [30,32,34] that used evaluators with no prior knowledge of the intervention. Coverage of the target population (pregnant women) by the intervention was *inadequate* in three studies.

### Intervention impact

Fourteen randomized studies involving a total of 307 018 women participants, with 292 256 live births, were included in the meta-analyses. Combined relative risks (RR) were calculated.

**Figure 2:**
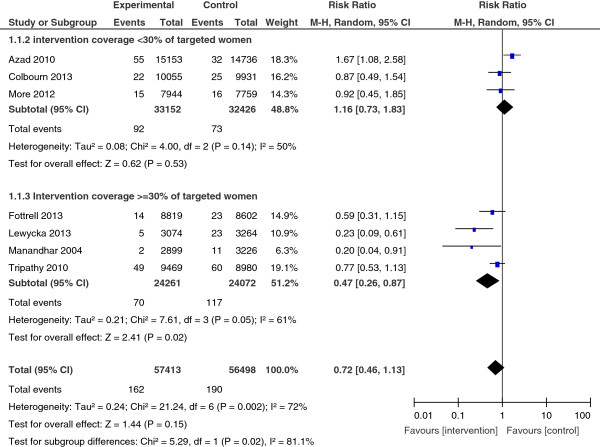
Maternal mortality, overall results and stratification by studies coverage.

*Maternal mortality* was measured in seven studies. When the results were combined, the reduction in maternal mortality was non-significant in the intervention groups (RR = 0.72; 95% CI: 0.46, 1.13). In addition, the seven results were heterogeneous (I^2^ = 72%, p = 0.002) (Figure 2).

A subgroup analysis of studies in which at least 30% of targeted women participated in interventions showed a 53% significant reduction in maternal mortality risk (four studies, RR = 0.47; 95% CI: 0.26, 0.87); with less heterogeity (I^2^ = 61%, p = 0.05).

**Figure 3:**
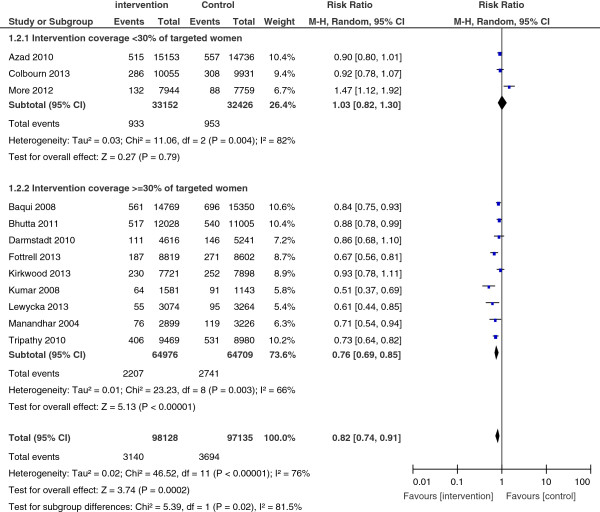
Neonatal mortality, overall results and stratification by studies coverage.

*Neonatal mortality* was measured in 12 studies. Their pooled results suggested a significant reduction of 18% neonatal mortality risk (RR = 0.82; 95% CI: 0.74, 0.91). But results were highly heterogeneous (I^2^ = 76%, p < 0.001) (Figure 3). A subgroup analysis of nine studies in which at least 30% of targeted women participated in interventions showed a statistically significant and greater reduction of up to 24% of neonatal mortality risk (RR = 0.76; 95% CI: 0.69, 0.85). However results remained heterogeneous (I^2^ = 66%, p = 0.003).

**Figure 4:**
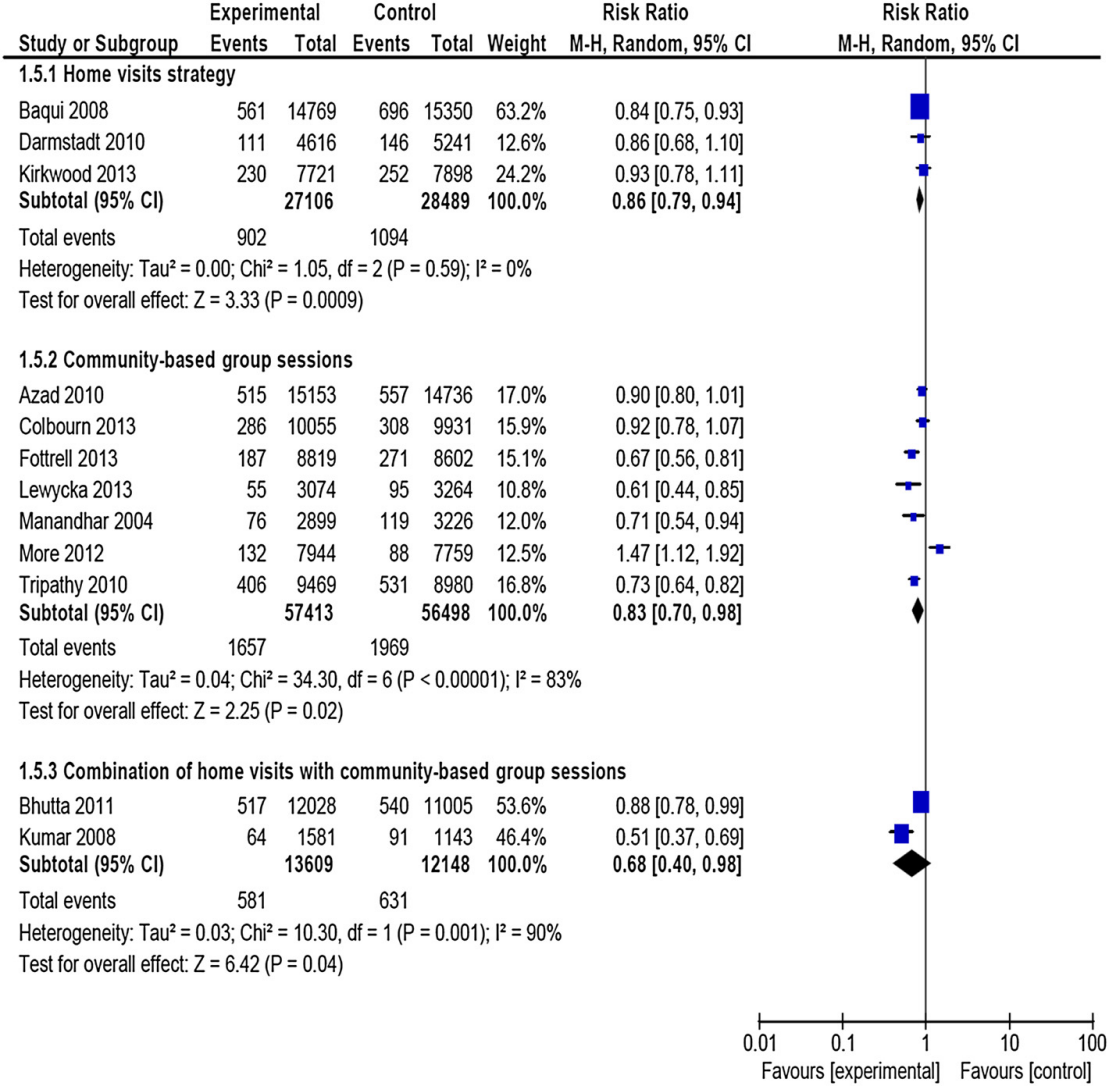
Neonatal mortality, subgroup analysis by type of intervention.

Stratified analyses suggested that the effects of the interventions differed depending on type of interventions. Two trials that combined home visits with community-based group sessions showed higher impact (RR = 0.68; 95% CI: 0.40, 0.98) than did those with either only home visits strategy (RR = 0.86; 95% CI: 0.79, 0.94) or only community-based group sessions (RR = 0.83; 95% CI: 0.70, 0.98) (Figure 4).

**Figure 5:**
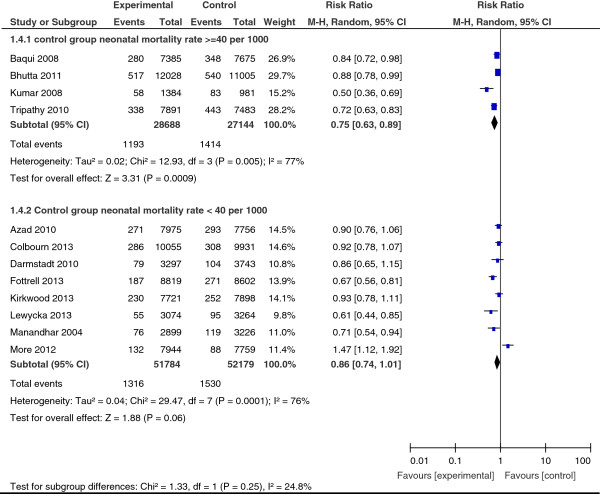
Neonatal mortality, subgroup analysis by level of mortality in control group.

In addition, the impact of the interventions fluctuated depending on the level of neonatal mortality observed in the control group (see Figure 5). In four trials in which the group registered a neonatal mortality rate of at least 40 per 1,000, the impact of the interventions was more marked, with a 25% significant reduction in risk of death (RR = 0.75; 95% CI: 0.63, 0.89). On the other hand, the reduction in risk was only 14% (RR = 0.86; 95% CI: 0.74, 1.01) in the other eight trials and did not reach statistical significance, where the neonatal mortality rate was under 40 per 1,000.

*Facility-based delivery*. Six studies were included in this analysis [26,30,33-36]. The aggregate result showed only a slight increase in the probability of facility-based delivery that was not statistically significant (RR = 1.16; 95% CI: 0.92, 1.45).

*Home delivery with skilled birth attendance*. Four studies measured the use of skilled birth attendance in home deliveries [20,26,32,33]. The combined effect of the interventions on this outcome was not statistically significant (RR = 1.06; 95% CI: 0.61, 1.85).

*Use of postpartum care*. This process outcome was measured in the two individual trials [29,30] conducted in urban settings. The effect of intervention was not significant.

*Conditional use of care in newborn illness*. Four studies evaluated this outcome [20,31-33]. The combined results indicated a substantial improvement in the probability of using skilled services among the reported cases of newborn illness (RR = 1.66; 95% CI: 1.23, 2.25). Stratified analysis showed no significant difference in relation to the educational strategy used.

*Clean cutting of the umbilical cord*. The use of sterile materials to cut the umbilical cord was measured for home deliveries in six studies [18,20,31-33]. The combined result showed a moderately statistically significant positive impact on this endpoint (RR = 1.33; 95% CI: 1.14, 1.55).

*Initiation of breastfeeding within one hour after birth*. This practice was also measured for home deliveries in four studies [18,31-33]. The aggregate effect of the interventions was positive, statistically significant and substantial in size (RR = 1.79; 95% CI: 1.27, 2.51).

*Knowledge of maternal and neonatal danger signs*/*birth preparedness and complications readiness*. We did not combine results for these two outcomes, because they were measured differently in the studies. Knowledge of danger signs was measured by two trials [30,31], and birth preparedness and complication readiness behaviours were also measured in two trials [29,32]. All studies showed improvements in measured outcomes.

## Discussion

We undertook this systematic review and meta-analysis to investigate the effectiveness of Birth Preparedness and Complications Readiness interventions in reducing maternal and neonatal morbidity and mortality and in improving process outcomes contributing to maternal and newborn survival. Fourteen randomized studies were selected for synthesis. The methodological quality of the studies was generally adequate except for criteria related to blinding of evaluators, since only three studies used evaluators who were blinded to the intervention.

### Key results of the review

The meta-analysis of 14 randomized studies showed that BPCR interventions were associated with significant reductions in neonatal mortality. Positive but statistically non-significant effects were shown for maternal survival. Significant improvements in some process outcomes associated with child survival (i.e., use of care in the event of newborn illness, clean cutting of the umbilical cord, and breastfeeding within the first hour after birth) were also shown.

In addition, two trials reported improvements in knowledge about danger signs, and two others [30,34] indicated that women in intervention groups were more likely to carry out birth preparedness and complication readiness activities than were their peers in the control groups.

### Interventions coverage of target population

Variation in the proportion of women reached by the interventions was an important factor in explaining heterogeneity of findings [21].

### Home visits versus women’s group sessions

Home visits and community-based women’s group sessions are both strategies that can potentially reduce the risk of neonatal mortality. However, subgroup analyses suggested that combining the two strategies would have a greater impact than would either one alone. While the number of studies may be insufficient to draw definitive conclusions, this observed tendency is logical as the two strategies are complementary. Home-based individual counselling is more personalized and appropriate for developing the mothers’ personal skills related to sanitary care practices. Community-based activities are still needed to support decision-making, because in traditional settings, decisions are more often taken by the community than by the individual. In practice, the choice of one strategy or another will depend on the social context and resource availability. Future studies that take into account cost parameters will be useful for comparing the different options.

### Regions with very high neonatal mortality rates

Subgroup analyses showed that reductions in neonatal mortality varied significantly depending on the neonatal mortality rate in the control group. The neonatal mortality risk decreased by 25% (RR = 0.75; 95% CI: 0.63, 0.89) in trials where the mortality rate in the control group was greater than 40 per 1,000. However, the decrease was not statistically significant in studies where the control group mortality rate was below 40 per 1,000. This result corroborates the hypothesis that educational interventions are more useful for preventing and managing infections [35]. In regions with high neonatal mortality (more than 40 per 1000), the cause of death structure is dominated by infectious diseases due to poor sanitation [16,35]. These conditions can be improved by implementing appropriate educational interventions that promote simple preventive measures [36]. On the other hand, in contexts where the neonatal epidemiological structure is dominated by non-infectious diseases (e.g. prematurity), educational interventions would seem to be less effective [31].

### Limitations of the review

The main limitation of this review was the small number of studies that were relevant for the investigation of our research questions. Subgroups analyses were undertaken with few studies. Several planned subgroup analyses could not be carried out because there were not enough studies covering the different modalities defined.

Furthermore, the results we obtained included important heterogeneities (expressed by the I^2^ statistic). Significant heterogeneities persisted in most of the subgroups for the stratified analyses.

### Interpretation of findings in the light of the scientific literature

Our results are consistent with those from two earlier reviews, while adding important complementary information. A 2010 review by Lassi and colleagues showed that community health workers and other health promotion agents could successfully implement important BPCR strategies such as home visits [19]. They found conclusive evidence of a reduction in neonatal mortality but inconclusive evidence of an effect on maternal mortality. A 2013 systematic review by Prost and colleagues focussing on the effects of women’s groups practising participatory learning and action found evidence of significant reductions in both neonatal and maternal mortality [21]. Our study confirms and extends these general findings in three ways. First, the BPCR concept is broader and more encompassing than the interventions studied in previous reviews, enabling consideration of a larger number of studies. As BPCR is widely used by governments, international agencies, and funding bodies, consideration of this broader concept is especially relevant for policy and practice. Second, this review was able to provide the first comparison of the relative value of specific BPCR components such as home visits, community mobilisation, and combined strategies. Third, our review is the first to examine results by level of neonatal mortality, providing insights into underlying mechanisms of disease causation and intervention effect. Together, these three systematic reviews underscore the potential value of several BPCR components in reducing maternal and neonatal mortality.

## Conclusions

### Implications for practice

There is evidence to support implementation of BPCR interventions to improve maternal and neonatal health in developing countries. Neonatal and maternal risks can be significantly reduced if home visits and/or women’s group sessions reach a high proportion of pregnant women. Decision-makers could support these approaches in settings where healthcare facilities are inadequate, where healthcare utilization is low, and where the burden of neonatal mortality is high. Sufficient resources should be mobilized for widespread implementation of these interventions and to ensure their quality, through ongoing training of educators/facilitators, provision of practice guidelines, and regular field supervision.

### Implications for research

Additional primary studies are needed to consolidate the results of our review. In particular, it will be important to conduct randomized trials of BPCR interventions in other regions with high maternal and neonatal risks. This is particularly important in francophone West and Central Africa, where no similar studies have as yet been conducted and the rates of neonatal and maternal mortality are among the highest in the world. It would be interesting to conduct trials of educational programs in certain areas where health facilities are sufficiently available but underused by the population [37].

The main methodological weakness that should be corrected in future trials relates to the blinding of evaluators. Blinding introduces special considerations in the context of cluster randomised trials [38]. Given that participants and implementation workers cannot generally be blinded to these types of interventions in cluster-randomised trials, it is important to use independent investigators to evaluate the outcomes.

Cost-effectiveness analyses are also needed to provide direction to decision-makers on the most efficient strategies to adopt. In addition, WHO has published prenatal standards of care, including prenatal education, with updates regarding developing birth and emergency plans to be applied in maternal services in developing countries [39]. It would be important to investigate the effectiveness of birth preparedness programs offered in routine prenatal clinics, where there is little control over the behaviours of the implementation workers or of the beneficiaries.

## Competing interests

The authors declare that they have no competing interests.

## Authors’ contributions

DS designed the study. DS conducted the study under the supervision of LG. DS and MJ extracted data from articles. DS and MH analyzed the data and wrote the initial draft of the manuscript. LG and MJ reviewed and critically revised the manuscript. All authors read and approved the final manuscript.

## Pre-publication history

The pre-publication history for this paper can be accessed here:

http://www.biomedcentral.com/1471-2393/14/129/prepub

## Supplementary Material

Additional file 1 Search strategy used for Embase database. Click here for file

Additional file 2 Description of studies included in the review. Click here for file

Additional file 3 Assessment of the methodological quality of studies. Click here for file
