# Supplementary material for: Birth Preparedness and Complication Readiness (BPCR) interventions to reduce maternal and neonatal mortality in developing countries: systematic review and meta-analysis
Source: BMC Pregnancy Childbirth. 2014 Apr 4;14:129. doi: 10.1186/1471-2393-14-129 (PMC4234142; doi:10.1186/1471-2393-14-129)
Supplement: Additional file 1 — Search strategy used for Embase database. [file 1471-2393-14-129-S1.docx]

*[*1. exp Parturition/

2. exp Delivery, Obstetric/

3. (birth* or childbirth* or delivery or deliveries or parturition* or obstetric*).ab,ti.

4. Developing Countries/

5. exp Asia/

6. exp Japan/

7. 5 not 6

8. exp Americas/

9. exp North America/

10. 8 not 9

11. exp Africa/

12. (readiness or plan* or prepar*).ab,ti.

13. 1 or 2 or 3

14. 4 or 7 or 10 or 11

15. 12 and 13 and 14

16. ((preparedness or readiness or plan or plans or planned or planning or preparation or

preparations or prepared) adj5 (birth or births or childbirth or childbirths or delivery or

deliveries or parturition or parturitions or obstetrics or obstetrical)).ab,ti.

17. 13 and 14 and 16

18. 15 or 17

19. limit 18 to (yr="1987 -Current" and (english or french) and (clinical trial, all or

clinical trial, phase i or clinical trial, phase ii or clinical trial, phase iii or clinical trial,

phase iv or clinical trial or comparative study or controlled clinical trial or evaluation

studies or meta analysis or multicenter study or randomized controlled trial or validation

studies))
