# Supplementary material for: Birth Preparedness and Complication Readiness (BPCR) interventions to reduce maternal and neonatal mortality in developing countries: systematic review and meta-analysis
Source: BMC Pregnancy Childbirth. 2014 Apr 4;14:129. doi: 10.1186/1471-2393-14-129 (PMC4234142; doi:10.1186/1471-2393-14-129)
Supplement: Additional file 2 — Description of studies included in the review. [file 1471-2393-14-129-S2.docx]

| **Authors and sittings** | **Design and participants** | **Intervention arms** | **Comparison arms** | **Outcome measures** |
| --- | --- | --- | --- | --- |
| Darmstadt et al  2010  Bangladesh | *Cluster- randomized study*  Pregnant women | Community health workers identified pregnant women; made two antenatal home visits to promote birth and newborn care preparedness; made four postnatal home visits to negotiate preventive care practices and to assess newborns for illness; and referred sick neonates to a hospital and facilitated compliance | Usual prenatal services in health organisations | antenatal and  immediate newborn care behaviours, knowledge of danger signs, care seeking for neonatal complications, and neonatal  mortality. |
| Mullany et al  2007  Nepal | Randomized trial  Pregnant women | *Intervention group 1*: Women received education with their husbands. The education intervention consisted of two 35-min health education sessions, including birth preparedness.  Sessions.  *Intervention group 2*: Women received the education intervention alone | Women received no education. | Birth preparedness behaviors, number of prenatal visits, institutional delivery, use of postnatal care. |
| Kumar et al  2008  India    (Uttar and Pradesh) | *Cluster- randomized study*  Pregnant women | Intervention group 1 : Community health workers did 2 prenatal home visits to deliver a preventive package of interventions for essential newborn care (birth preparedness, clean delivery and cord  care, thermal care [including skin-to-skin care], breastfeeding promotion, and danger sign recognition);  Intervention group 2 :  women received the package of essential newborn care provided to group 1, plus use of a liquid crystal hypothermia indicator (ThermoSpot). | Usual services of governmental and non-governmental organisations in the area | Neonatal mortality, perinatal mortality. Use of antenatal care, tetanus vaccination, behavior birth preparation (identification of skilled attendant, structure of care, financial arrangements, baby items), place of birth, use of skilled attendant, hygienic care practice for newborn and breastfeeding practice. |
| Baqui et al.  2008  Bangladesh. | *Cluster- randomized study*  *married women of reproductive age* | *Individual home care arm*: female community health workers (one per 4000 population) identified pregnant women, made two antenatal home visits to promote birth and newborn-care preparedness, made postnatal home visits to assess newborns on the first, third, and seventh days of birth, and referred or treated sick neonates  *Group community-care arm:* birth and newborn-care preparedness and skilled care seeking were promoted solely through group sessions held by female and male community counsellors. | Routine services provided in health structures | Neonatal mortality, use of prenatal care, taking iron supplements, tetanus vaccination, hygienic practices for neonatal care (own instrument section of the umbilicus, first bath delayed, initiated breastfeeding in the first hour) |

| **Authors and sittings** | **Design and participants** | **Intervention arms** | **Comparison arms** | **Outcome measures** |
| --- | --- | --- | --- | --- |
| Manandhar et al (2004)  Nepal | *Cluster- randomized study*  Pregnant women | - Perinatal healthcare structures strengthening  - A female facilitator convened nine women's group meetings every month. The facilitator supported groups through an action-learning cycle in which they identified local perinatal problems and formulated strategies to address them. | Perinatal healthcare structures strengthening | Neonatal mortality, maternal mortality, stillbirths, uptake of antenatal and delivery services, home-care practices at delivery and postpartum, infant morbidity, health care seeking. use of prenatal care, facility delivery, use of a skilled birth attendant, home-based care practices for the newborn, initiation of breastfeeding within the first hour of life |
| Tripathy et al  2010  India | *Cluster- randomized study*  Pregnant women | - Creation of local health committee to participate in efforts to improve the quality of maternal and neonatal care.  - a facilitator convened 13 groups every month to support participatory action and learning for women, and facilitated the development and implementation of strategies to address maternal and newborn health problems. | Creation of local health committee to participate in efforts to improve the quality of maternal and neonatal care. | Neonatal mortality rate, maternal depression score, stillbirths, maternal and perinatal deaths, use of prenatal care, facility delivery, uptake of antenatal and delivery services, home-based care practices during and after delivery, and health-care seeking behaviour, exclusive breastfeeding |
| Azad et al  2010  Bangladesh | *Cluster- randomized study*  Pregnant women | - Health services strengthening and basic training of traditional birth attendants.  - A facilitator convened 18 groups every month to support participatory action and learning for women, and to develop and implement strategies to address maternal and neonatal health problems. | Health services strengthening and basic training of traditional birth attendants. | Neonatal mortality, maternal mortality, stillbirths, uptake of antenatal and delivery services, home-care practices during and after delivery, infant morbidity, health-care seeking behaviour, perinatal mortality, and early and late neonatal mortality rate, initiation of breastfeeding within the first hour of life. |
| Belizan et al  1995  Cuba, Brazil, Argentina and Mexico. | *randomized trial*  Pregnant women | A home intervention of four to six visits dealing with psychosocial support and education about health-related habits, alarm signs, hospital facilities, antismoking and anti-alcohol programs, and a reinforcement of adequate health services utilization for the pregnant woman and a support person. | Routine prenatal care | Knowledge of obstetrical alarm signs and symptoms, knowledge of signs of labor onset, changes in dietary, smoking and alcohol consumption practices, use of prenatal and postnatal care |

| **Authors and sittings** | **Design and participants** | **Intervention arms** | **Comparison arms** | **Outcome measures** |
| --- | --- | --- | --- | --- |
| More et al, 2012  India | *Cluster- randomized study*  Pregnant women | 24 clusters  Women’ groups followed a cycle of 36 meeting in the communities, led by facilitator (local woman with leadership skills) | 24 clusters | Primary: stillbirths, neonatal mortality rate and extended perinatal mortality rate, perinatal care, and maternal morbidity  Secondary: maternal mortality ratio, antenatal  care, institutional delivery, breastfeeding, and careseeking for newborn illness |
| Lewycka et al, 2013  Malawi | *Cluster- randomized study*  Cohort of women aged 10-49years who delivered a child between Feb 1, 2006 and Jan 31, 2009 | 24 clusters, 9374 births  Women’s groups were supported by a female facilitators through a participatory learning and actions cycle of 20 meetings | 24 clusters | Primary: neonatal, perinatal, and infant mortality rates, and maternal mortality ratio  Secondary: maternal and infant morbidity, use of skilled maternity services, immunisation, malaria prophylaxis, use of prevention  of mother-to-child transmission services, and  breastfeeding |
| Colboun et al, 2013  Malawi | *Cluster- randomized study*  An open cohort of pregnant womwn | 15 clusters  81 volunteer facilitators formed women’ groups that followed a participatory learning and action cycle to improve maternal and neonatal health | 17 clusters | Primary: maternal mortality ratio, and perinatal, and neonatal mortality rates  Secondary: institutional delivery, percentage of  maternal deaths subjected to audit, case fatality rates, practice of signal obstetric care functions |
| Fottrel et al, 2013  Bengladesh | *Cluster- randomized study*  Pregnant women | Nine clusters  - Health services strengthening and basic training of traditional birth attendants.  - A facilitator convened groups every month to support participatory action and learning for women, and to develop and implement strategies to address maternal and neonatal health problems. | Nine clusters  Health services strengthening and basic training of traditional birth attendants. | Primary: neonatal mortality rate  Secondary: stillbirth, perinatal mortality rate, pregnancy related mortality, institutional delivery, home-care practices, and health-care seeking |

| **Authors and sittings** | **Design and participants** | **Intervention arms** | **Comparison arms** | **Outcome measures** |
| --- | --- | --- | --- | --- |
| Kirkwood et al, 2013  Ghana | *Cluster- randomized study*  Pregnant women | 49 zones  Community-based surveillance volunteers make two home visits during pregnancy three in the first week of life to promote essential newborn-care practices | 49 zones | Neonatal mortality, initiation of breastfeeding, care-seeking, four or more antenatal-care visits, money saved for delivery or emergency |
| Bhutta et al, 2011  Pakistan | *Cluster- randomized study*  Pregnant women | 8 clusters  The intervention package delivered by lady health workers (LHWs) through home visitations and group sessions consisted of promotion of antenatal care and maternal health education, use of clean delivery kits, facility births, immediate newborn care, identification of danger signs, and promotion of careseeking; | 8 clusters  control clusters received routine care. | Neonatal mortality, Stillbirths, Women delivering in a fa Mothers initiating breastfeeding within 30 minutes; Home deliveries using a clean delivery kit. |
