# Supplementary material for: Birth Preparedness and Complication Readiness (BPCR) interventions to reduce maternal and neonatal mortality in developing countries: systematic review and meta-analysis
Source: BMC Pregnancy Childbirth. 2014 Apr 4;14:129. doi: 10.1186/1471-2393-14-129 (PMC4234142; doi:10.1186/1471-2393-14-129)
Supplement: Additional file 3 — Assessment of the methodological quality of studies. [file 1471-2393-14-129-S3.docx]

| **Studies** | **Randomization** | **Allocation sequence concealment** | **Blinding of outcome assessment** | **Contamination** | **Co-intervention** | **Coverage** | **Quality of implementation** | **Loss to follow up** | **Intention to treat Analysis** |
| --- | --- | --- | --- | --- | --- | --- | --- | --- | --- |
| Manandhar et al (2004) | A | A | Inadequate | A | A | A | A | A | A |
| [Azad al (2010](#_ENREF_5)) | A | A | Inadequate | A | A | Inadequate | A | A | A |
| [Tripathy et al (2010](#_ENREF_136)) | A | A | Inadequate | A | A | A | A | A | A |
| [Baqui et al (2008](#_ENREF_8)) | A | A | Inadequate | Inadequate | A | A | A | A | A |
| [Darmstadt et al (2010](#_ENREF_23)) | A | A | UC | A | A | A | A | A | A |
| [Kumar et al (2008](#_ENREF_73)) | A | A | A | A | A | A | A | A | A |
| [Mullany et al (2007](#_ENREF_93)) | A | A | Inadequate | A | A | A | A | A | A |
| [Belizan et al (1995](#_ENREF_14)) | A | A | A | A | A | A | A | A | A |
| Colbourn et al (2013) | A | A | Inadequate | A | A | Inadequate | A | A | A |
| Fottrell et al (2013) | A | A | Inadequate | A | A | A | A | A | A |
| Kirkwood et al (2013) | A | A | Inadequate | A | A | A | A | A | A |
| Lewycka et al (2013) | A | A | Inadequate | A | A | A | A | A | A |
| More et al (2012) | A | A | Inadequate | A | A | Inadequate | A | A | A |
| Bhutta et al (2011) | A | A | A | A | A | A | A | A | A |
